# Scurvy among the Troops

**Published:** 1900-09-01

**Authors:** 


### SCURVY AMONG THE TROOPS.

A paper from the pen of Professor A. E. Wright, of Netley, appeared in last week's *Lancet*, which is worthy of attention for other reasons besides the point raised by the author. Professor Wright's point is that scurvy is due to the food containing a considerable excess of mineral acids over bases, and that the proper prophylaxis and treatment of the condition consists in the administration of salts of oxidisable organic acids, such as the lactate of soda, inasmuch as such treatment would lead by the most direct means to the retention or restoration of the normal alkalinity of the blood. All this, however, by the way. Just at the present moment the interest in these cases lies in the fact that such cases of scurvy should be met with in men from Ladysmith arriving at Netley in May and June. Most of the cases had gone through the siege of Ladysmith, where they had no doubt suffered quite enough to give them scurvy a dozen times over, but when one reads the detailed description of the miserable plight in which these poor fellows arrived at Netley, and the apparently active condition in which their scurvy still remained so long after the cessation of the hardships to which they had been exposed, one can but ask first whether these patients had ever been fit to put on board a transport, and, next, what sort of rations they had had during the voyage?

